# Antinociceptive Effects of Lipid Raft Disruptors, a Novel Carboxamido-Steroid and Methyl β-Cyclodextrin, in Mice by Inhibiting Transient Receptor Potential Vanilloid 1 and Ankyrin 1 Channel Activation

**DOI:** 10.3389/fphys.2020.559109

**Published:** 2020-09-23

**Authors:** Ádám Horváth, Tünde Biró-Sütő, Boglárka Kántás, Maja Payrits, Rita Skoda-Földes, Eszter Szánti-Pintér, Zsuzsanna Helyes, Éva Szőke

**Affiliations:** ^1^Department of Pharmacology and Pharmacotherapy, Medical School, University of Pécs, Pécs, Hungary; ^2^János Szentágothai Research Centre and Centre for Neuroscience, University of Pécs, Pécs, Hungary; ^3^Department of Organic Chemistry, Institute of Chemistry, University of Pannonia, Veszprém, Hungary

**Keywords:** inflammation, lipid rafts, methyl β-cyclodextrin, nerve terminal, pain, sensory neuron, steroid, Transient Receptor Potential channel

## Abstract

Transient Receptor Potential Vanilloid 1 and Ankyrin 1 (TRPV1, TRPA1) cation channels are expressed in nociceptive primary sensory neurons, and play an integrative role in pain processing and inflammatory functions. Lipid rafts are liquid-ordered plasma membrane microdomains rich in cholesterol, sphingomyelin, and gangliosides. We earlier proved that lipid raft disintegration by cholesterol depletion using a novel carboxamido-steroid compound (C1) and methyl β-cyclodextrin (MCD) significantly and concentration-dependently inhibit TRPV1 and TRPA1 activation in primary sensory neurons and receptor-expressing cell lines. Here we investigated the effects of C1 compared to MCD in mouse pain models of different mechanisms. Both C1 and MCD significantly decreased the number of the TRPV1 activation (capsaicin)-induced nocifensive eye-wiping movements in the first hour by 45% and 32%, respectively, and C1 also in the second hour by 26%. Furthermore, C1 significantly decreased the TRPV1 stimulation (resiniferatoxin)-evoked mechanical hyperalgesia involving central sensitization processes, while its inhibitory effect on thermal allodynia was not statistically significant. In contrast, MCD did not affect these resiniferatoxin-evoked nocifensive responses. Both C1 and MCD had inhibitory action on TRPA1 activation (formalin)-induced acute nocifensive reactions (paw liftings, lickings, holdings, and shakings) in the second, neurogenic inflammatory phase by 36% and 51%, respectively. These are the first *in vivo* data showing that our novel lipid raft disruptor carboxamido-steroid compound exerts antinociceptive and antihyperalgesic effects by inhibiting TRPV1 and TRPA1 ion channel activation similarly to MCD, but in 150-fold lower concentrations. It is concluded that C1 is a useful experimental tool to investigate the effects of cholesterol depletion in animal models, and it also might open novel analgesic drug developmental perspectives.

## Introduction

Transient Receptor Potential (TRP) Vanilloid 1 and Ankyrin 1 (TRPV1 and TRPA1) cation channels are multisteric receptors activated by a variety of inflammatory mediators and tissue irritants, temperature changes and mechanical stimuli besides the classical exogenous agonists such as capsaicin (CAPS), resiniferatoxin (RTX) and formaldehyde, allyl-isothiocyanate (in mustard oil), respectively (McKemy et al., 2002; Peier et al., 2002; Reid and Flonta, 2002; Grimm et al., 2003, 2005; Lee et al., 2003; Bandell et al., 2004; Corey et al., 2004; Jordt et al., 2004; Macpherson et al., 2005, 2007; McNamara et al., 2007; Trevisani et al., 2007; Wagner et al., 2008; Majeed et al., 2010; Vilceanu and Stucky, 2010; Vriens et al., 2011, 2014; Bautista et al., 2013; Drews et al., 2014; Oberwinkler and Philipp, 2014). TRPV1 and TRPA1 are often co-localized on the CAPS-sensitive peptidergic sensory neurons and play key regulatory roles in pain and inflammation (Szolcsányi, 2004; Salas et al., 2009). Pro-inflammatory neuropeptides such as Substance P and calcitonin gene-related peptide released from the activated CAPS-sensitive sensory nerve fibers evoke vasodilation, plasma protein extravasation and inflammatory cells activation in the innervated area called neurogenic inflammation, as well as nociceptor sensitization (Helyes et al., 2003a, 2009; Szolcsányi, 2004). Therefore, both TRPV1 and TRPA1 have been in the focus of analgesic and anti-inflammatory drug development, especially for the treatment of chronic neuropathic pain and inflammatory diseases with neurogenic inflammatory components (chronic obstructive pulmonary diseases, psoriasis, arthritis, inflammatory bowel diseases) (Moran et al., 2011; Kaneko and Szallasi, 2014; Nilius and Szallasi, 2014). The presently available drugs do not provide satisfactory pain relief in most cases or induce severe side effects after long-term use (Botz et al., 2017). Great efforts have been put into the development of TRPV1 antagonists which proved to be very effective in both preclinical and phase II and III clinical trials, but due to their hyperthermic side effects they could not be registered in the clinical practice (Helyes et al., 2003b; Lee et al., 2015). TRPA1 is also considered to be a promising analgesic target based on experimental and human studies which seem to be free of severe side effects (Romanovsky et al., 2009; Botz et al., 2017). These data clearly suggest the drug developmental potential of TRPV1 and TRPA1 blockade, therefore alternative mechanisms in addition to the direct antagonism have been proposed as promising inhibitors options (Ferrari and Levine, 2015; Sághy et al., 2015, 2018; Lin et al., 2019).

Recent results of extensive lipid raft research in the last two decades have had a great impact on cell biology and pharmacology. Lipid rafts are specialized microdomains in the plasma membrane rich in cholesterol, sphingomyelins and gangliosides (Simons and Ikonen, 1997). Several receptors, ion channels and signaling molecules including TRPV1 and TRPA1 ion channels are located in lipid rafts and disruption of these membrane regions affects their functions (Liu et al., 2006; Morenilla-Palao et al., 2009; Szőke et al., 2010; Sághy et al., 2015). However, data are controversial about the outcomes of lipid raft modulation on TRP channels. Although several *in vitro* data show that lipid raft decomposition inhibits TRP channel opening, there are only two recent *in vivo* evidence. Methyl β-cyclodextrin (MCD)-induced membrane cholesterol depletion led to antinociception in the RTX-evoked mononeuropathy model via phosphatidylinositol 4,5-bisphosphate (PI(4,5)P2) hydrolysis (Lin et al., 2019) and significantly attenuated the prostaglandin E2 (PGE2)-evoked mechanical hyperalgesia in rats (Ferrari and Levine, 2015).

Several endogenous steroids have been described to inhibit TRPV1. Dehydroepiandrosterone (DHEA) is able to decrease CAPS-evoked currents in primary sensory neurons (Chen et al., 2004). However, it is not clear if DHEA bind directly to the CAPS-binding domain or it is an allosteric modulator of TRPV1. The neurosteroid pregnenolone sulfate (PS) has a variety of neuropharmacological actions including glycinergic synaptic transmission in the pain pathway. PS, pregnanolone, pregnanolone sulfate, progesterone or dihydrotestosterone administration in extracellular way significantly inhibited TRP Canonical 5 (TRPC5) channel activation within 1–2 min, 17β-estradiol (E2) and dehydroepiandrosterone sulfate had weak inhibitory effects. TRPC5 channels are able to direct stereo-selective steroid modulation quickly, and it is lead to channel inhibition (Majeed et al., 2011). We published earlier that our novel synthetic carboxamido-steroid compound (C1) decreased activation of TRP channels located on primary sensory neurons, such as TRPV1, TRPA1, TRP Melastatin 3 (TRPM3), and TRP Melastatin 8 (TRPM8). Furthermore, we provided the first evidence and the presence and the position of the carboxamido group was important for this action mediated by cholesterol depletion from the plasma membrane. This effect was similar to that of MCD, but in a much lower, 1000-fold concentration (Szánti-Pintér et al., 2015; Sághy et al., 2018).

Based on these data obtained on primary sensory neuronal cultures here we investigated the effects of C1 compound in mouse pain models of different mechanisms related to TRPV1 and TRPA1 activation in comparison with MCD.

## Materials and Methods

### Animals and Ethics

Twelve to sixteen week-old male C57BL/6 mice were used to test CAPS-evoked nocifensive reactions, and NMRI mice of the same age and sex in the formalin-, and RTX-induced models. The animals were kept in standard plastic cages at 24–25°C, under a 12–12 h light-dark cycle and provided with standard rodent chow and water *ad libitum* in the Laboratory Animal House of the Department of Pharmacology and Pharmacotherapy, University of Pécs. All experimental procedures were carried out according to the 1998/XXVIII Act of the Hungarian Parliament on Animal Protection and Consideration Decree of Scientific Procedures of Animal Experiments (243/1988). The studies were approved by the Ethics Committee on Animal Research of Pécs University according to the Ethical Codex of Animal Experiments and license was given (license No.: BAI/35/702-6/2018.).

### Synthesis of Steroid Compound C1

The steroid compound C1 was synthesized by a method, which was described earlier in details (Horváth et al., 2011; Szánti-Pintér et al., 2011, 2015). In brief, the 16-keto-18-nor-13α-steroid was obtained via an unusual Wagner–Meerwein rearrangement of 16α,17α-epoxy-5α-androstane in the presence of an imidazolium-based ionic liquid (Horváth et al., 2011). The derivatization of the unnatural steroid was performed by Barton’s methodology leading to an iodoalkene mixture (Szánti-Pintér et al., 2015). The iodoalkene mixture was converted to N-(prop-2-ynyl)-carboxamides via a palladium-catalyzed aminocarbonylation reaction and after a column chromatography, C1 was obtained in pure form.

### CAPS-Evoked Acute Chemonocifensive Reaction

The effects of C1 and MCD compared to the saline control were investigated on acute chemonociception, 30 μg/ml CAPS (20 μl) was instilled in the right eye of the mice. Local pretreatments (20 μl) with 100 μM C1 or 15 mM MCD were performed 30 min before the test. CAPS-induced eye-wiping movements with the forelegs were counted during 1 min, as previously described (Szolcsányi et al., 1975; Szöke et al., 2002). We counted only the one-leg movements, washing- or other two-hand movements were excluded. CAPS instillation was repeated 2 and 3 h after its first administration.

### RTX-Induced Thermal Allodynia and Mechanical Hyperalgesia

Resiniferatoxin (0.1 μg/ml, 20 μl, ultrapotent TRPV1 agonist) was injected intraplantarly into right hindpaws. RTX induces an acute neurogenic inflammation with rapid development of thermal allodynia due to peripheral sensitization, and later mechanical hyperalgesia due to both peripheral and central mechanisms (Meyer and Campbell, 1981; Pan et al., 2003). Control thermo- and mechanonociceptive thresholds were measured on two consecutive days before the experiment, which were used for self-control comparisons. Intraplantar pretreatments (20 μl) with 100 μM or 500 μM C1 and 15 mM MCD were performed 30 min before the RTX administration, which evokes a short acute nocifensive reaction of paw licking, biting, lifting or shaking. The thermonociceptive threshold was measured by an increasing temperature Hot Plate (IITC Life Science, Woodland Hills, CA, United States) 10, 20, and 30 min after RTX injection, and the mechanical hyperalgesia was investigated by Dynamic Plantar Aesthesiometer (DPA, Ugo Basile, Italy) 30, 60, and 90 min after RTX administration, as described earlier (Almási et al., 2003; Payrits et al., 2017; Kántás et al., 2019).

### Formalin-Evoked Acute Nocifensive Behavior

Intraplantarly injected formalin (20 μl, 2.5%) immediately induced nocifensive reactions. The duration of hindpaw licking, lifting, shaking and holding in an elevated position were measured between 0 and 5 min (first phase). It is related to direct chemical stimulation of nociceptors mainly via the TRPA1 receptor. After a resting period (ca. 10–15 min), the duration of the nocifensive behaviors was measured between 20 and 45 min (second phase). This is due to neurogenic inflammatory mechanisms (Tjølsen et al., 1992). Intraplantar pretreatments (20 μl) with 100 μM C1 or 15 mM MCD were performed 30 min before formalin administration.

### Drugs and Chemicals

Methyl β-cyclodextrin (Sigma, St. Louis, MO, United States) was dissolved in saline to reach final concentration of 15 mM (500 mg/kg). C1 was dissolved in dimethyl sulfoxide to obtain a 10 mM stock solution. Further dilution was made with saline to reach final concentrations of 100 μM (850 μg/kg) or 500 μM (4.25 mg/kg). CAPS from Sigma was diluted with saline from a 10 mg/ml stock solution of 10% ethanol, 10% Tween 80 in saline to reach final concentration of 30 μg/ml. RTX (Sigma) was dissolved in ethanol to yield a 1 mg/ml stock solution, and further diluted with saline to reach final concentration of 0.1 μg/ml. Formalin was diluted with phosphate-buffered saline from a 6% formaldehyde stock solution (Molar Inc., Hungary).

### Statistical Analysis

All data are the means ± SEM of six animals per group in the CAPS-evoked eye wiping test and formalin test, and 12–20 animals per group in the RTX-induced thermal allodynia and mechanical hyperalgesia model. Statistical analysis was performed by Two-way ANOVA followed by Bonferroni’s multiple comparisons *post hoc* test, in all cases *p* < 0.05 was considered statistically significant.

## Results

### C1 and MCD Reduce the Number of CAPS-Evoked Eye-Wiping Movements

**FIGURE 1:**
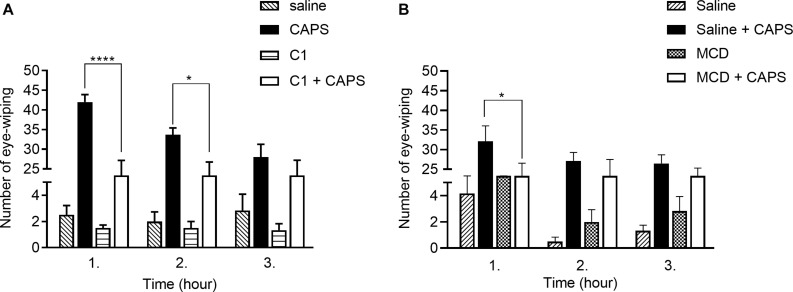
Effect of 100 μM C1 **(A)** and 15 mM MCD **(B)** in the CAPS-evoked acute chemonociceptive reaction. Both compounds reduced the number of eye-wipings. Data are means ± SEM of *n* = 6 animals/group. Two-way ANOVA with Bonferroni *post hoc* test was used for statistical analysis (*p < 0.05; ****p < 0.0001 C1/MCD pretreatment vs. saline pretreatment).

The number of CAPS-evoked eye-wipings in the 1st, 2nd, and 3rd h in the saline-pretreated group were 42.0 ± 1.9; 33.7 ± 1.8; 28.0 ± 3.3, respectively. In contrast, the corresponding values in the C1-pretreated group were: 23.0 ± 4.2; 23.0 ± 3.8; 23.7 ± 3.5 (Figure 1A). In case of MCD pretreatment, the number of CAPS-evoked wiping in the saline-pretreated control animals were 32.2 ± 3.9; 27.2 ± 2.1 and 26.5 ± 2.2 after 1st, 2nd, and 3rd CAPS instillation. MCD-pretreated animals showed less of eye-wipings with the following results: 23.8 ± 2.7; 24.2 ± 3.3; 22.7 ± 2.7 (Figure 1B).

In both cases slightly decreasing response to CAPS was observed due to CAPS-evoked desensitization of the TRPV1 receptor. C1 significantly and gradually decreased the number of eye-wipings both in the 1st and 2nd h, while MCD exerted significant effect only in the 1st h.

### C1 and MCD Do Not Influence RTX-Induced Thermal Allodynia

The baseline heat threshold values of untreated mice were between 44°C and 49°C. RTX-induced 9.5–16.3 ± 2.3–3.1%; 9.1–9.6 ± 2.3–3.2% and 4.3–5.3 ± 1.4–1.6% (39.0–41.9 ± 1.1–1.5°C; 41.8–42.3 ± 0.8–1.4°C; 43.9–44.5 ± 0.6–1.0°C) drop of the thermonociceptive threshold 10, 20, and 30 min after its intraplantar injection in the saline-pretreated control groups. The corresponding values were 11.6 ± 2.3% (40.7 ± 1.1°C); 3.3 ± 1.6% (43.9 ± 0.9°C); 3.8 ± 1.6% (47.0 ± 0.5°C) for 100 μM C1, 14.5 ± 1.8% (39.7 ± 0.9°C); 6.6 ± 1.4 (43.3 ± 0.6°C); 0.3 ± 1.7% (46.5 ± 0.6°C) for 500 μM C1 and 8.3 ± 2.0% (42.4 ± 1.0°C); 7.8 ± 0.9% (42.6 ± 0.3°C); 5.8 ± 2.1% (43.5 ± 1.2°C) for 15 mM MCD.

**FIGURE 2:**
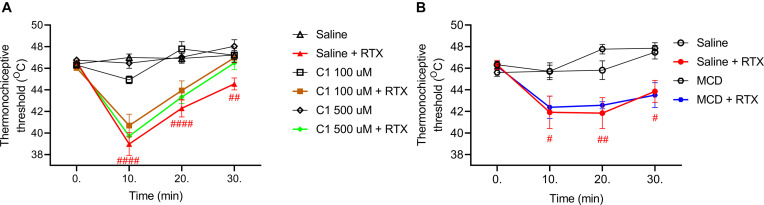
Effect of 100 μM or 500 μM **(A)** and 15 mM MCD **(B)** on the RTX-induced thermal allodynia. Neither lower or higher concentration of C1, nor MCD did not influence the thermonociceptive threshold changing. Red lines represent the saline pretreatment, brown or green lines the 100 μM or 500 μM C1 pretreatment and blue line the 15 MCD pretreatment, respectively. Data are means ± SEM of *n* = 12–20 animals/group. Red hashes represent the significance in the saline-pretreated group (values after RTX-injection compared to control). Two-way ANOVA with Bonferroni *post hoc* test was used for statistical analysis.

Neither C1 nor MCD altered the RTX-induced thermal allodynia (Figures 2A,B).

### C1 Diminishes RTX-Induced Mechanical Hyperalgesia

The basal mechanonociceptive thresholds of the intact mouse paw were between 8 and 10 g. RTX-evoked drop of the mechanonociceptive threshold values were 43.9–44.5 ± 3.2–6.2%; 37.3–37.9 ± 3.9–8.1% and 26.9–39.5 ± 3.0–4.2% (5.0–5.4 ± 0.3–0.6 g; 5.6–6.0 ± 0.4–0.7 g; 5.5–7.0 ± 0.2–0.4 g) 30, 60, and 90 min after the injection in the saline pretreated control groups. The corresponding values were 30.0 ± 5.2% (6.5 ± 0.4 g); 20.1 ± 5.0% (7.4 ± 0.4 g); 10.4 ± 4.8% (8.3 ± 0.4 g) for 100 μM C1, 19.0 ± 3.1% (7.8 ± 0.3 g); 16.6 ± 2.9% (8.0 ± 0.3 g); 14.3 ± 3.0% (8.2 ± 0.8 g) for 500 μM C1 and 36.6 ± 6.4% (5.4 ± 0.5 g); 43.4 ± 3.4% (4.9 ± 0.3 g); 29.7 ± 6.4% (6.0 ± 0.5 g) for 15 mM MCD.

**FIGURE 3:**
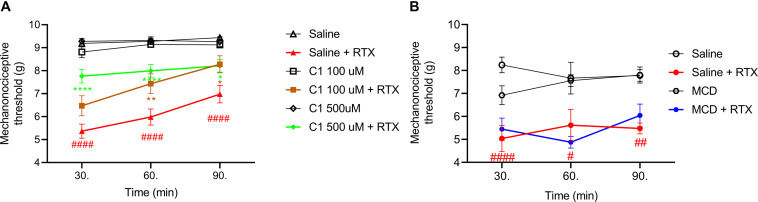
Effect of 100 μM or 500 μM **(A)** and 15 mM MCD **(B)** on the RTX-induced mechanical hyperalgesia. Both 100 μM and 500 μM C1 alleviated, while MCD did not altered the mechanonociceptive threshold changing. Red lines represent the saline pretreatment, brown or green lines the 100 μM or 500 μM C1 pretreatment and blue line the 15 MCD pretreatment, respectively. Data are means ± SEM of *n* = 12–20 animals/group. Red hashes represent the significance in the saline-pretreated group (values after RTX-injection compared to control). Two-way ANOVA with Bonferroni *post hoc* test was used for statistical analysis (*p < 0.05; **p < 0.01; ****p < 0.0001 C1 pretreatment vs. saline pretreatment).

Both 100 μM and 500 μM of C1 alleviated the RTX-induced mechanical hyperalgesia, but MCD had no effect (Figures 3A,B).

### C1 and MCD Alleviate Formalin-Evoked Acute Nocifensive Behaviors

**FIGURE 4:**
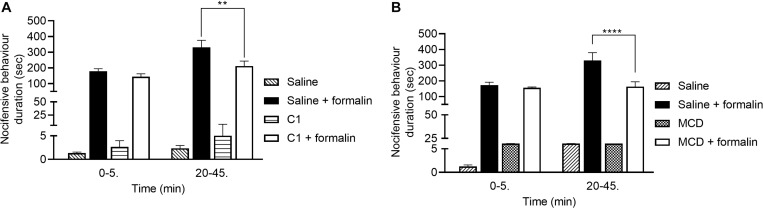
Effect of 100 μM C1 **(B)** and 15 mM MCD **(A)** in the formalin-evoked acute nocifensive behaviors. Both C1 and MCD altered the second, neurogenic inflammatory phase. Data are means ± SEM of *n* = 6 animals/group. Two-way ANOVA with Bonferroni *post hoc* test was used for statistical analysis (**p < 0.01; ****p < 0.0001 C1/MCD pretreatment vs. saline pretreatment).

The durations of formalin-evoked acute nocifensive behaviors in the saline-pretreated control group were 179.5 ± 16.0 s and 331.5 ± 45.0 s in the first and second phases, respectively. In the C1 pretreated animals these values were 144.2 ± 18.5 s and 212.2 ± 31.5 s (Figure 4A). In case of MCD pretreatment, the nocifensive behaviors durations in the saline control group were 173.9 ± 17.6 s and 330.5 ± 49.2 s in the two phases, respectively. Compared to the MCD-pretreated group, the corresponding values were 155.9 ± 5.1 s and 163.2 ± 31.3 s (Figure 4B).

Neither C1 nor MCD modified the nocifensive behaviors in the first phase related to direct chemical activation of TRPA1 receptors, but both compounds significantly decreased the pain reactions in the second phase resulting from acute neurogenic inflammation.

## Discussion

We present here the analgesic effect of lipid raft decomposition depleting cholesterol by C1 and MCD (Szánti-Pintér et al., 2015; Sághy et al., 2018). We demonstrated that C1 and MCD diminished TRPV1 and TRPA1 activation-induced acute nocifensive behaviors, furthermore, C1 inhibited the development of TRPV1 stimulation-evoked mechanical hyperalgesia.

Both C1 and MCD significantly diminished the number of CAPS instillation-induced eye-wiping movements in the 1st h by 45 and 32%, respectively, and C1 also in the 2nd h by 26%. We observed a slightly decreasing response in the 2nd and 3rd h to CAPS due to desensitization of TRPV1 receptor (Sharma et al., 2013). Furthermore, C1 significantly decreased RTX-induced mechanical hyperalgesia involving central sensitization processes as well, while its inhibitory effect on thermal allodynia induced predominantly by peripheral sensitization mechanisms (Pan et al., 2003) was not statistically significant. In contrast MCD did not affect these RTX-induced nocifensive responses. Both compounds had inhibitory action on formalin-evoked acute neurogenic inflammatory nocifensive reactions (paw liftings, lickings, holdings, and shakings) in the second, neurogenic inflammatory phase by 36 and 51%, respectively.

These novel *in vivo* results are well supported by our previous *in vitro* findings showing that C1 and MCD significantly and concentration-dependently inhibit TRPV1 and TRPA1 receptor activation both on primary sensory neuronal cultures and receptor-expressing cell line (Szőke et al., 2010; Szánti-Pintér et al., 2015; Sághy et al., 2015, 2018). We have previously proved by filipin staining and fluorescence spectroscopy that C1 similarly to MCD depleted cholesterol from the plasma membrane of sensory neurons, and therefore, they are both considered to be lipid raft disruptors (Sághy et al., 2018). Furthermore, we described that the presence and the position of the carboxamido group on the steroidal skeleton are substantial for TRP channel inhibition. The importance of stereoselectivity was emphasized for the inhibitory effects of steroids on the TRPC5 cation channel. Progesterone and pregnanolone diminished TRPC5 channel function, while the stereo-isomer of pregnanolone, pregnenolone and a progesterone metabolite allopregnanolone had no inhibitory effects. It is suggested, that stereo-isomerism due to a minimal structural change might be sufficient to alter the biological effect (Majeed et al., 2011). CAPS-induced currents in sensory neurons were decreased by DHEA, but the molecular mechanism is unclear. Although the authors suggested its direct effects on the CAPS-binding domain or an allosteric modulation its action on the lipid rafts surrounding the TRPV1 is also possible (Chen et al., 2004). In a previous study we demonstrated that E2 incubation anticipated the TRPV1 desensitization via the tropomyosin-related kinase A (TrkA) receptor. We provided *in vivo* and *in vitro* evidence for E2-induced TRPV1 receptor sensitization mediated by TrkA via E2-evoked genomic and non-genomic mechanisms (Payrits et al., 2017). Cholesterol depletion by MCD decreased the CAPS-evoked currents in dorsal root ganglion (DRG) primary sensory neurons (Liu et al., 2006). In contrast, MCD did not influence the heat-evoked responses on TRPV1-transfected *Xenopus laevis* oocytes (Liu et al., 2003) or ^3^H-RTX binding to TRPV1 receptors on rat C6 glioma cells (Bari et al., 2005). Cholesterol enrichment in isolated membrane segments can modulate the temperature threshold for TRPV1 activation through specific Cholesterol Recognition/interaction Amino acid Consensus (CRAC) motifs (Morales-Lázaro and Rosenbaum, 2019). Increased membrane cholesterol, but not its diastereoisomer epicholesterol addition, inhibited CAPS-, heat- and voltage-induced TRPV1 currents (Picazo-Juárez et al., 2011). These results were also supported by structural studies of CRACs (Levitan et al., 2014; Saha et al., 2017).

Although there are several *in vitro* evidence that lipid raft disruption affected TRP channel activation (Szőke et al., 2010; Sághy et al., 2015), there are only sporadic, recent *in vivo* reports. MCD-related cholesterol depletion induced antinociception in RTX-induced mononeuropathy through PI(4,5)P2 hydrolysis in mice (Lin et al., 2019). Intraplantar injection of MCD attenuated the PGE2-, but not cyclopentyladenosine-evoked mechanical hyperalgesia. It is suggest that the development of PGE2-evoked hyperalgesia is closely related to lipid raft integrity (Ferrari and Levine, 2015). Both local and systemic administration of random methylated β-cyclodextrins (RAMEB) attenuated complete Freund’s adjuvant-induced thermal allodynia and mechanical hyperalgesia in rats. RAMEB might capture the prostaglandin content and then decrease the inflammatory pain which might be a novel anti-inflammatory and analgesic tool (Sauer et al., 2017). Intraplantar injection of another components of lipid rafts, the ganglioside GT1b, produced nociceptive responses and enhanced formalin-induced nocifensive reactions. On the other hand, intraplantar injection of sialidase, which cleaves sialyl residues from gangliosides, attenuated these responses (Watanabe et al., 2011; Sántha et al., 2020). The flavanone isosakuranetin blocked PS-induced Ca^2+^-influx in DRG neurons and significantly attenuated the noxious heat- and PS-induced pain sensation in mice (Straub et al., 2013).

The present *in vivo* data provide the first evidence that the novel C1 compound modifying lipid rafts surrounding the TRPV1 and TRPA1 ion channels exerts antinociceptive and antihyperalgesic effects. The maximal inhibitory effect observed in both TRPV1 and TRPA1 activation-induced nocifensive tests were similar to that of MCD, but in 150-fold lower concentrations. Furthermore, C1 proved to be effective also on RTX-evoked mechanical hyperalgesia that was not affected by MCD. However, despite the well-established lipid rafts disrupting abilities of both C1 and MCD (Szőke et al., 2010; Sághy et al., 2015, 2018), their direct inhibitory actions on the TRPV1 and TRPA1 ion channel activation cannot be excluded.

We conclude that the novel C1 compound is a useful experimental tool to investigate the effects of cholesterol depletion in animal models, and it also might open novel opportunities for analgesic drug development.

## Data Availability Statement

All datasets presented in this study are included in the article/supplementary material.

## Ethics Statement

The animal study was reviewed and approved by the Ethics Committee on Animal Research, University of Pécs.

## Author Contributions

ÉS and JSz contributed to the conceptualization. MP, ÁH, BK, TB-S, ESP, and RS-F contributed to the methodology. ÁH and TB-S contributed to the formal analysis, writing – original draft preparation, visualization, and project administration. ÁH, BK, TB-S, and MP contributed to the investigation. ZH and ÉS contributed to the resources, writing – review and editing, supervision, and funding acquisition. All authors contributed to the article and approved the submitted version.

## Conflict of Interest

The authors declare that the research was conducted in the absence of any commercial or financial relationships that could be construed as a potential conflict of interest.

## Abbreviations

C1: carboxamido-steroid compound
CAPS: capsaicin
CRAC: Cholesterol Recognition/interaction Amino acid Consensus
DHEA: dehydroepiandrosterone
DRG: dorsal root ganglion
E2: 17- β estradiol
MCD: methyl β-cyclodextrin
PGE2: prostaglandin E2
PI(4,5)P2: phosphatidylinositol 4,5-bisphosphate
PS: pregnenolone sulfate
RAMEB: random methylated β-cyclodextrins
RTX: resiniferatoxin
TrkA: tropomyosin-related kinase A
TRP: Transient Receptor Potential
TRPA1: Transient Receptor Potential Ankyrin 1
TRPC5: Transient Receptor Potential Canonical 5
TRPM3: Transient Receptor Potential Melastatin 3
TRPM8: Transient Receptor Potential Melastatin 8
TRPV1: Transient Receptor Potential Vanilloid 1.
